# A comparative study of the Au-catalyzed cyclization of hydroxy-substituted allylic alcohols and ethers

**DOI:** 10.3762/bjoc.7.91

**Published:** 2011-06-14

**Authors:** Berenger Biannic, Thomas Ghebreghiorgis, Aaron Aponick

**Affiliations:** 1 Department of Chemistry, University of Florida, P.O. Box 117200, Gainesville, FL 32611, U.S.A

**Keywords:** allylic alcohol, allylic ether, Au-catalyzed, S_N_2', tetrahydropyran

## Abstract

The Au(I)-catalyzed cyclization of hydroxyallylic ethers to form tetrahydropyrans is reported. Employing (acetonitrile)[(*o*-biphenyl)di-*tert*-butylphosphine]gold(I) hexafluoroantimonate, the cyclization reactions were complete within minutes to hours, depending on the substrate. The reaction progress was monitored by GC, and comparisons between substrates demonstrate that reactions of allylic alcohols are faster than the corresponding ethers. Additionally, it is reported that Reaxa QuadraPure^TM^ MPA is an efficient scavenging reagent that halts the reaction progress.

## Introduction

Saturated oxygen heterocycles are found in a wide variety of biologically interesting and structurally complex natural products [1]. These compounds are typically densely functionalized and contain numerous stereogenic centers. Many challenges for the total synthesis of these molecules revolve around issues of selectivity and can be complicated by the presence of sensitive functional groups. While cyclization reactions of highly elaborated substrates are desirable, mild chemoselective methods are necessary for this endeavor.

**Scheme 1:**
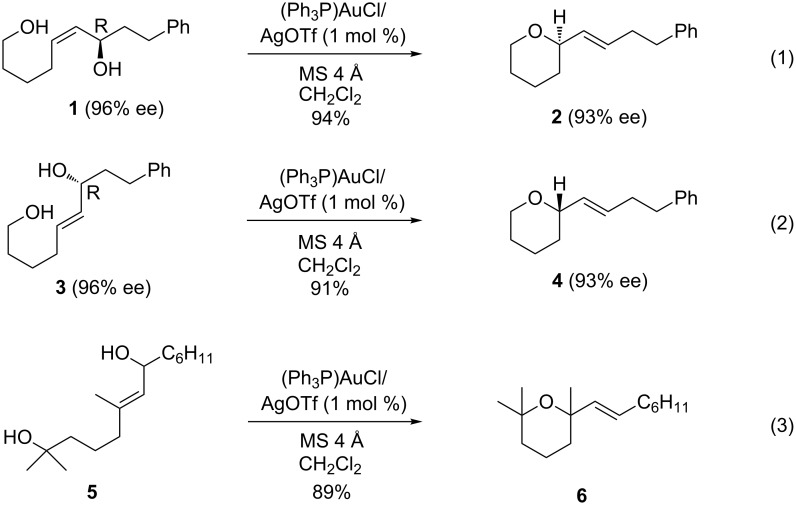
Au-catalyzed cyclization reactions of monoallylic diols.

Homogeneous gold-catalyzed reactions have emerged as a powerful new methodology for the construction of a diverse array of molecular architectures; for recent reviews on Au-catalysis, see [2–10]. Generally, only mild conditions are necessary and these processes are highly chemoselective. While the typical substrates employed in these reactions effect transformations on alkyne, allene, and alkene moieties, recent reports from our laboratory and others have demonstrated that unsaturated alcohols, such as allylic and propargylic alcohols, are reactive substrates that readily participate in dehydrative formal S_N_2' reactions [11–25]. The formation of tetrahydropyrans is easily accomplished with monoallylic diol substrates, as illustrated in Scheme 1 [23–25]. The reactions are generally rapid and high yielding with low catalyst loading and can be carried out at low reaction temperatures. Additionally, they are stereospecific, as changing the olefin geometry provides enantiomeric products (Scheme 1, reaction 1 versus reaction 2) [25], and they are also tolerant of highly substituted substrates (Scheme 1, reaction 3) [23].

Although these features are attractive from a synthetic point of view, one potential disadvantage is that both the nucleophile and electrophile are alcohols that may require the introduction and cleavage of protecting groups in the preparation of more complex substrates. In the course of a synthetic project, we required a monoallylic diol but encountered difficulty due to an errant protecting group scheme. It was surmised that the problem would be solved if the allylic alcohol leaving group did not need to be revealed directly before the cyclization event. This led us to consider the use of alternative substrates where the allylic alcohol could be deprotected under the reaction conditions, or the use of other “protecting groups” that would also serve as leaving groups and obviate the need for a separate deprotection step. We reasoned that the best group to introduce would be one that was not susceptible to cleavage by standard deprotection conditions and therefore would only be removed after the desired cyclization reaction. Since alcohols are usually very poor leaving groups, but function extremely well in the present system, it seemed likely that a fairly robust group could perform satisfactorily here. Additionally, calculations suggest that in intermolecular hydroalkoxylation reactions of allenes the kinetic allylic ether products are isomerized by Au(I)-NHC complexes to the regioisomeric thermodynamic (and observed) products [26]. Successful implementation of such a strategy would offer an alternative to the use of the highly successful and well-established set of leaving groups employed in π-allylmetal chemistry [27–32]. Herein we report a study of Au-catalyzed cyclizations with different leaving groups that do not require deprotection, and data on the reaction progress that allows comparison between leaving groups and *cis*- versus *trans*-olefins.

## Results and Discussion

At the outset, one of the important goals was to be able to make comparisons between how well different substrates function in the reaction. Previous papers detail the results with diols and include a variety of substrates with yields and reaction times [23–25]. While the isolated yield is the ultimate measure of how well the system has performed, these data do not provide sufficient details to compare accurately between different classes of substrates. We also sought to gain more insight into how fast the reaction proceeds and to be able to comment on catalyst lifetime.

**Scheme 2:**
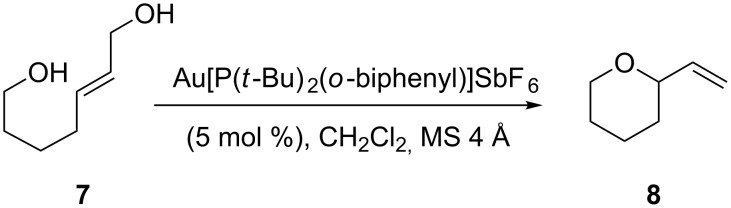
Reaction conditions for the preparation of **8**.

To be consistent, we chose to study the simple system shown in Scheme 2 and to vary the nature of the allylic leaving group and olefin geometry. These conditions are slightly different to those employed in the study of diols [23–25], differing in catalyst identity and loading (1 mol % (Ph_3_P)AuCl/AgOTf versus 5 mol % Au[P(*t*-Bu)_2_(*o*-biphenyl)]SbF_6_).

**Figure 1:**
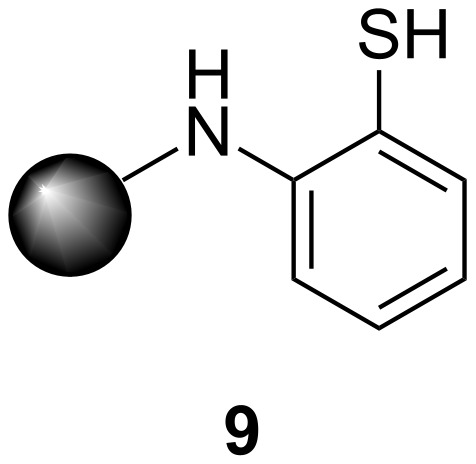
QuadraPure^TM^ MPA.

As mentioned above, the ability to follow the reaction progress was desired but this presented several practical challenges. As the reactions are often complete within minutes, a continuous method of analysis would be necessary, or alternatively samples could be collected over the course of the reaction with analysis to follow. Initial experiments focused on using ^1^H NMR, but this raised concerns due to the heterogeneous nature of the reaction mixture, which contained molecular sieves to absorb the water that was generated. Analysis by GC with decane as an internal standard was then explored. A standard curve was prepared with **8**, but, due to the fact that Au-complexes are fairly stable in air and moisture, a quenching method was needed to obtain accurate results. In a typical experiment, the reaction is generally filtered through a short plug of silica, but for small aliquots (25 μL) this was not practical. Instead the resin bound scavenging agent QuadraPure^TM^ MPA **9** (Figure 1) was employed.

**Figure 2:**
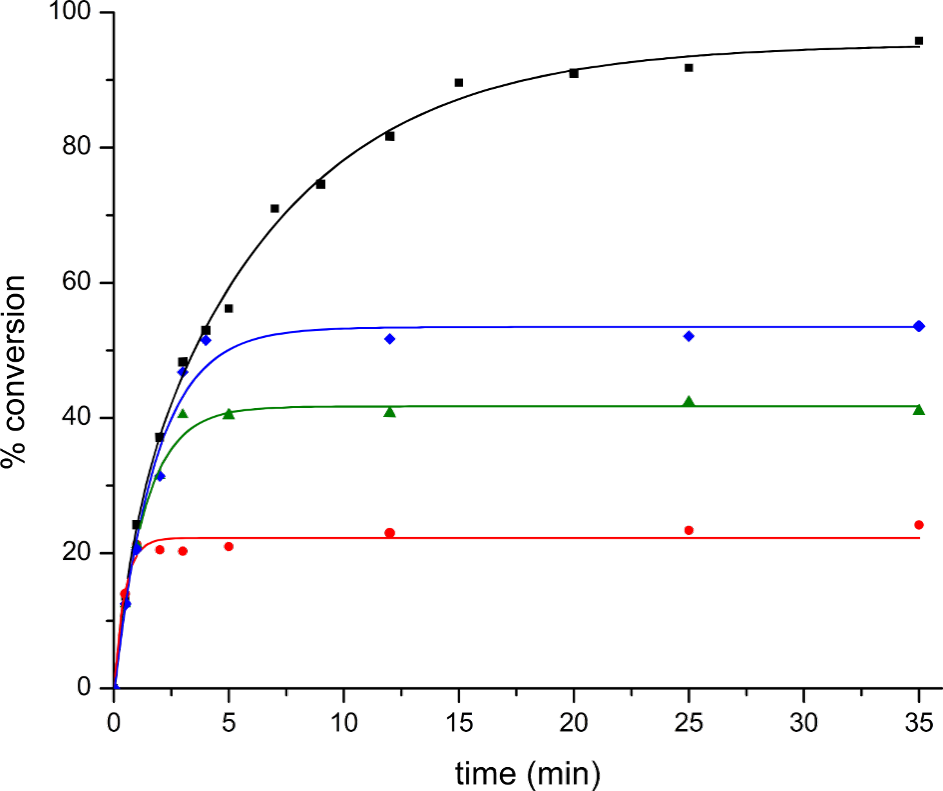
Quenching experiments using **9**. 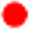 = quenched after 1 min; 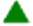 = quenched after 3 min; 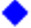 = quenched after 5 min.

To the best of our knowledge, scavenging reagents such as this have not previously been employed in homogeneous Au-catalysis and it was necessary to validate this method. In a typical reaction, the goal was to quench aliquots by injecting them into vials containing **9** suspended in CH_2_Cl_2_. For a detailed protocol see the Supporting Information File 1. As a test, samples were continually taken from the reaction illustrated in Scheme 2 and treated with **9** until TLC analysis indicated that the reaction was complete. GC analysis of the samples provided the data used to construct the black curve shown in Figure 2, which shows the expected behavior and was reproducible. As a control experiment, a sample taken after 3 minutes was diluted with CH_2_Cl_2_, but not exposed to the resin. After 16 h, the reaction had proceeded to 95% conversion demonstrating that **9** is necessary to halt the progress of the reaction. The precision of the analysis also warrants comment. At several points throughout the reaction, the same sample was analyzed 5 times. In each of these sample sets, the range of percent conversion spanned approximately 2%. The standard deviation from the mean was 0.92%.

Figure 2 also demonstrates that the reactions can be quenched with QuadraPure^TM^ MPA. The curves in red, green, and blue show the results of reactions that were quenched after 1, 3, and 5 minutes, respectively. The reaction conditions were otherwise identical to the reaction shown in black, which proceeded to 96% conversion, while the reactions quenched at 1, 3, and 5 minutes went to 23%, 41%, and 52% conversion, respectively.

**Figure 3:**
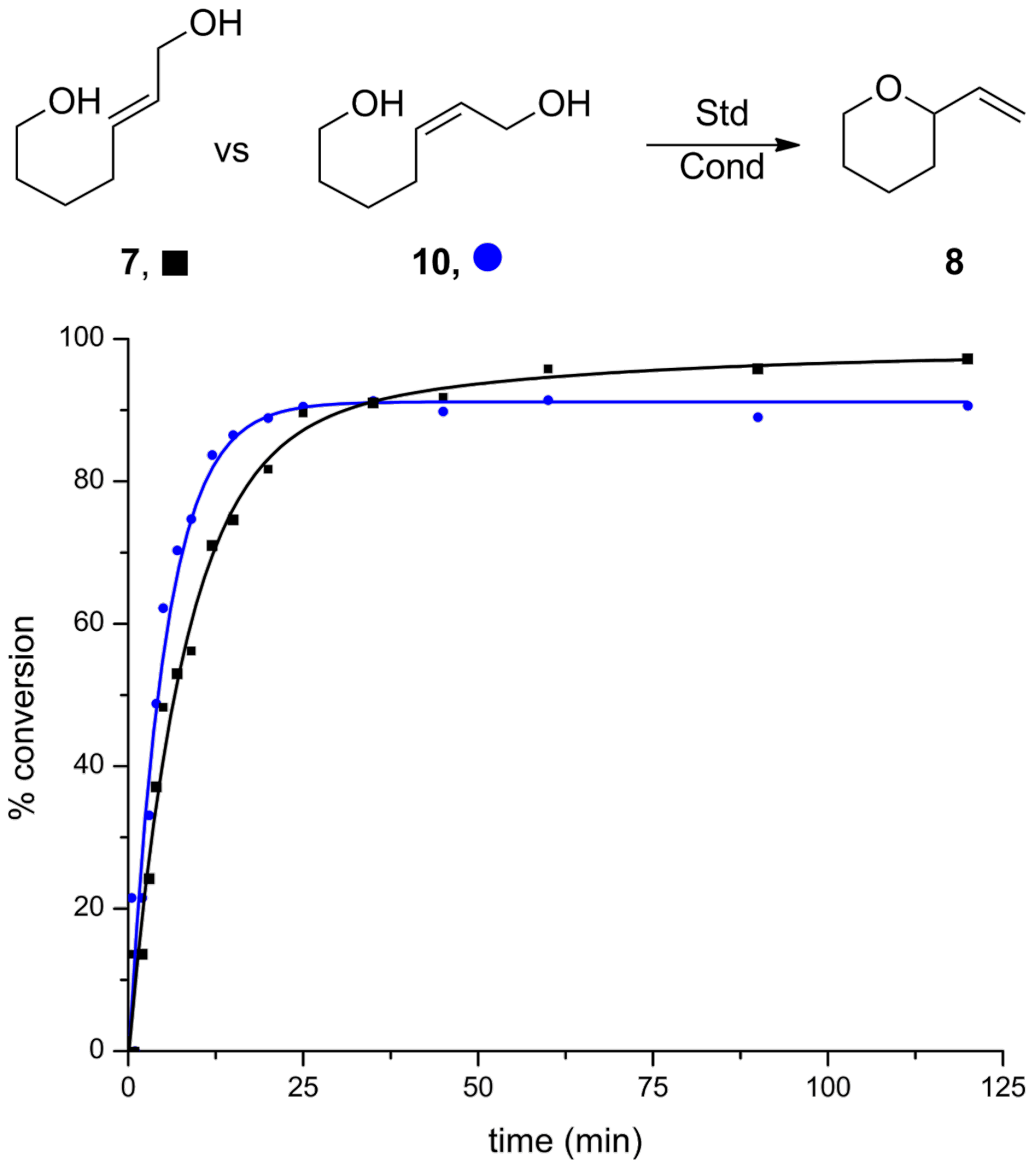
Reaction progress in *cis*- and *trans*-diols. Conditions: 5 mol % Au[P(*t*-Bu)_2_(*o*-biphenyl)]SbF_6_, CH_2_Cl_2_, MS 4 Å.

Subsequently, a comparison between *cis*- and *trans*-diols **10** and **7** was made. As can be seen in Figure 3, both reactions were fairly rapid, with the *cis*-diol **10** being only slightly faster in the initial period than **7**. Interestingly, the reaction of **7** achieves higher conversion overall, but both substrates have >90% conversion after 25 minutes.

**Figure 4:**
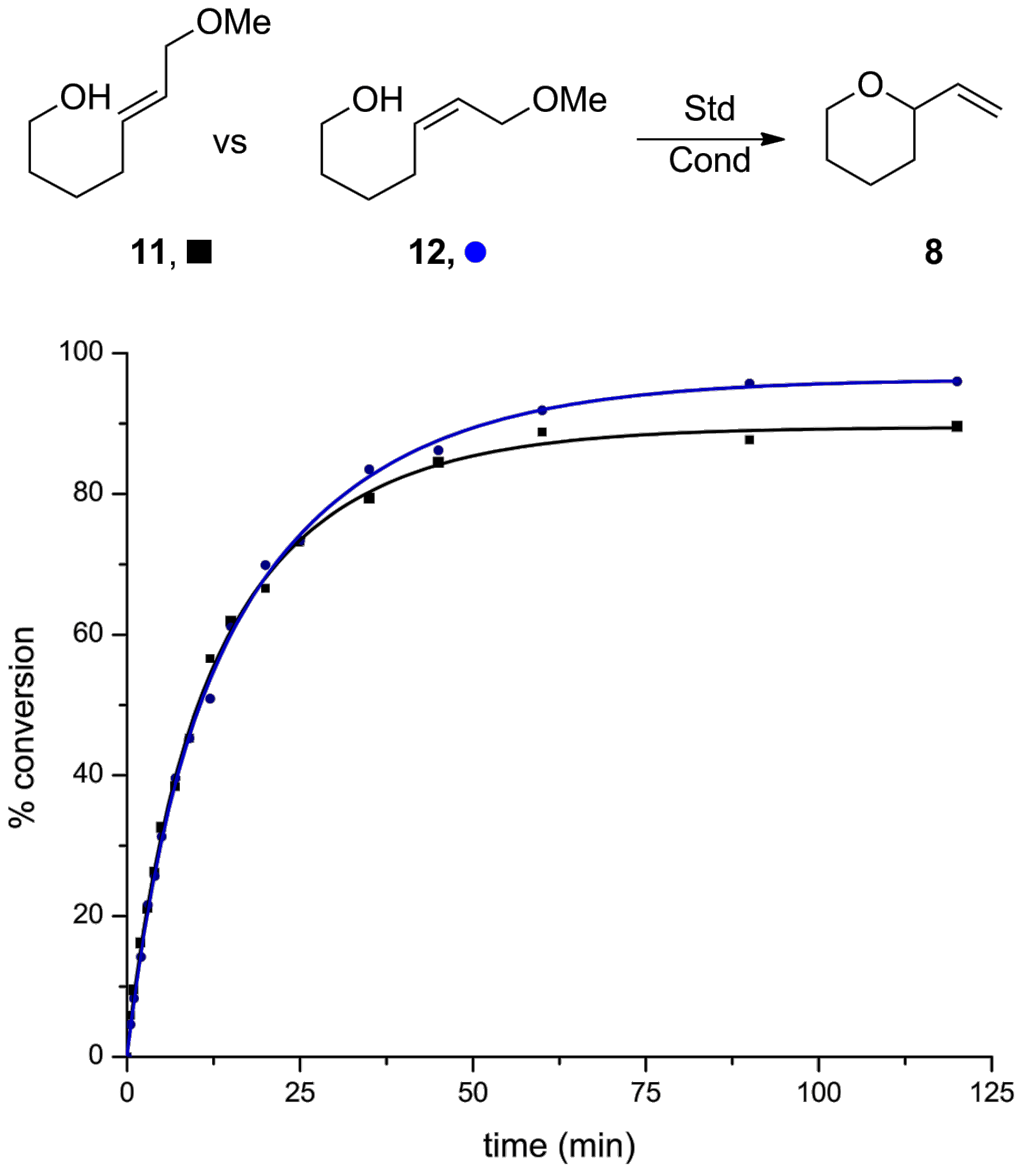
Reaction progress in *cis*- and *trans*-methyl ethers. Conditions: 5 mol % Au[P(*t*-Bu)_2_(*o*-biphenyl)]SbF_6_, CH_2_Cl_2_, MS 4 Å.

The methyl ethers **11** and **12** were explored and proved to be suitable substrates (Figure 4). While these reactions were slightly slower in the initial stages than the corresponding diols, excellent conversions were achieved. This demonstrates that methyl ethers fit the criteria described above. The methyl group efficiently shields this functional group under a variety of commonly used conditions and it can then act as a leaving group under Au-catalyzed cyclization conditions.

**Figure 5:**
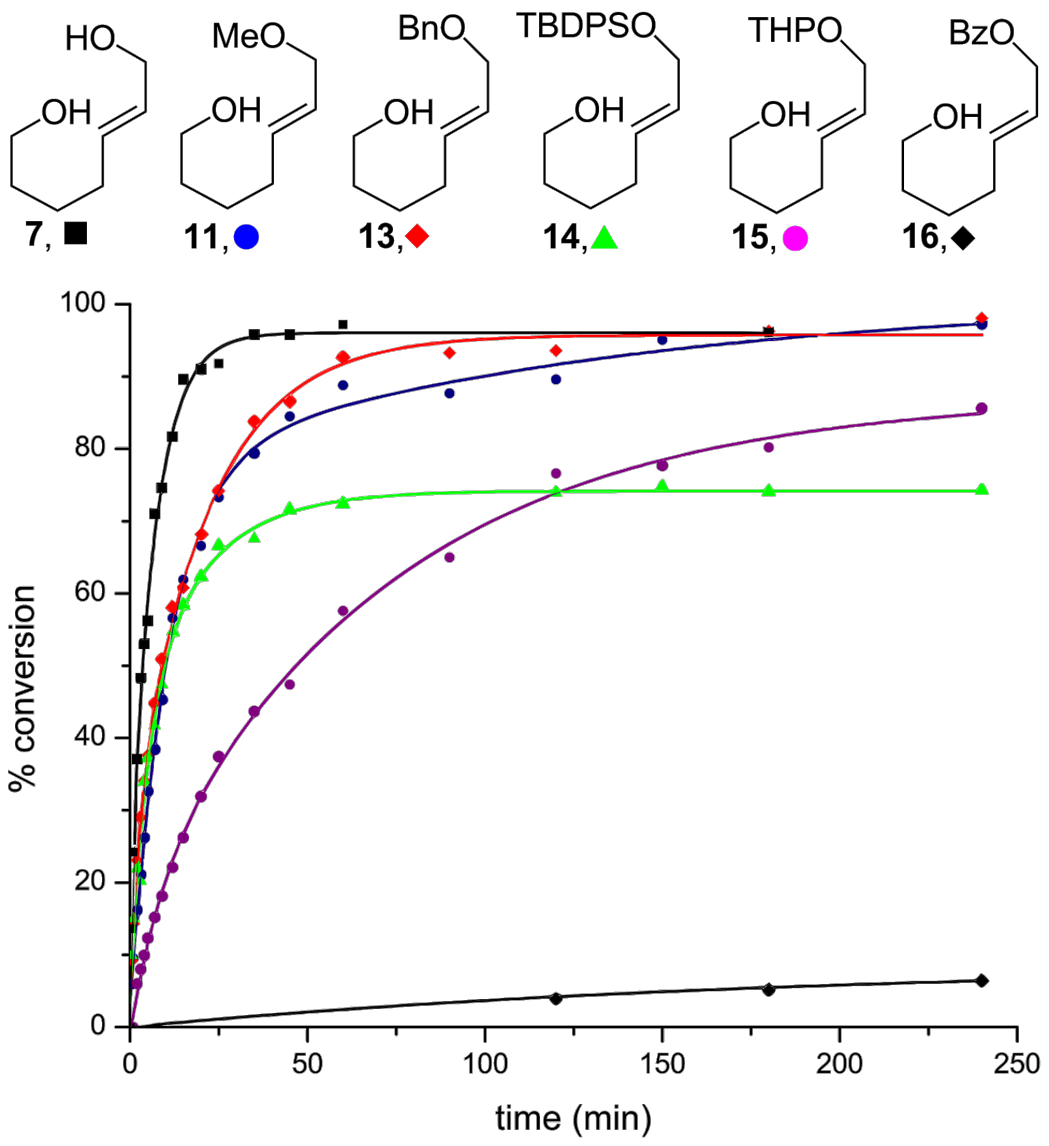
A comparison of commonly used protecting groups. Conditions: 5 mol % Au[P(*t*-Bu)_2_(*o*-biphenyl)]SbF_6_, CH_2_Cl_2_, MS 4 Å.

Several additional, commonly used, protecting groups were also screened under the reaction conditions (Figure 5). From the graph, it is apparent that benzyl (**13**), TBDPS (**14**), and THP [33] (**15**) could all be used, but esters such as benzoyl (**16**) were unsuitable. This may provide a basis for chemoselective transformations, as allyl esters are readily ionized by Pd^0^ complexes and the resulting π-allylpalladium species are alkylated by a variety of nucleophiles [27–28].

**Figure 6:**
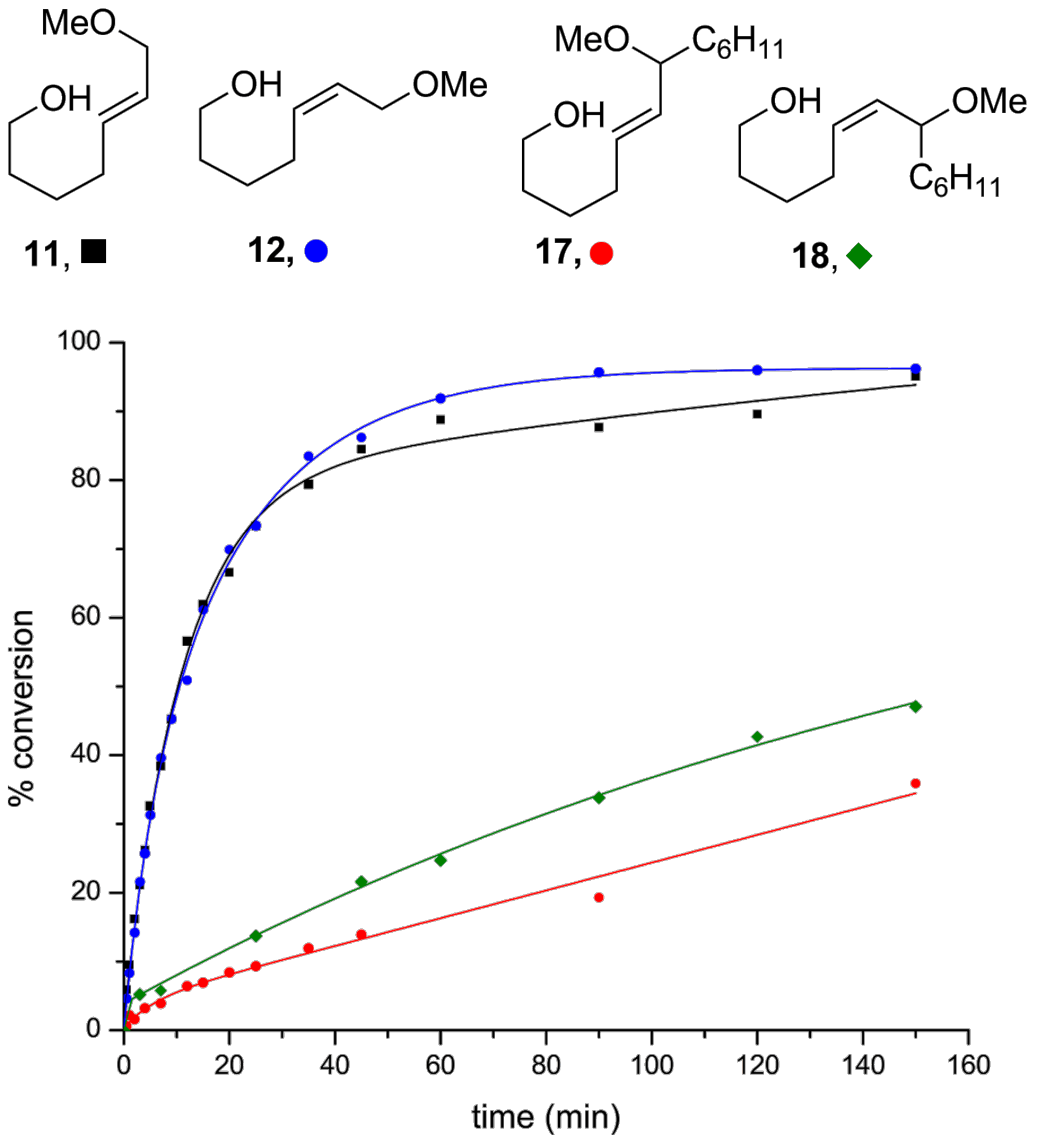
Comparison of 1° and 2° allylic ethers. Conditions: 5 mol % Au[P(*t*-Bu)_2_(*o*-biphenyl)]SbF_6_, CH_2_Cl_2_, MS 4 Å.

Finally, 1° allylic and 2° allylic ether substrates were compared (Figure 6). Substitution at the allylic position drastically slows the reaction. Although the conversion of **17** and **18** is low on the timescale shown, the reactions continue and after 48 h provide acceptable, but moderate yields. The corresponding *trans*- and *cis*-cyclohexyl-substituted diols (not shown) provide the products in 96% and 92% isolated yields after 40 minutes, respectively [23–25]. While cyclohexyl substituents significantly slow the reaction, it is likely that other less sterically demanding substituents will be better tolerated. This is currently under investigation with more synthetically useful substrates.

## Conclusion

In conclusion, it has been demonstrated that a variety of allylic ethers undergo Au-catalyzed formal S_N_2' reactions to form tetrahydropyrans. The reaction of allylic alcohols appears to be faster, although the leaving group is traditionally not considered to be as good. Reactions of *cis*-substrates appear to be slightly faster than the corresponding *trans*-allylic ethers. While the difference is small, it suggests that it is better to prepare the *cis*-substrates, and this is also very straightforward via a number of different routes. Further studies on secondary allylic ethers and on the application of the method in total synthesis are ongoing and will be reported in due course.

## Supporting Information

File 1 General procedures and characterization data for all new compounds.
