# Supplementary material for: A comparative study of the Au-catalyzed cyclization of hydroxy-substituted allylic alcohols and ethers
Source: Beilstein J Org Chem. 2011 Jun 14;7:802–7. doi: 10.3762/bjoc.7.91 (PMC3135158; doi:10.3762/bjoc.7.91)
Supplement: File 1 — General procedures and characterization data for all new compounds. [file Beilstein_J_Org_Chem-07-802-s001.pdf]

Supporting Information

for

A comparative study of the Au-catalyzed cyclization of hydroxy-substituted allylic alcohols and ethers

Berenger Biannic, Thomas Ghebreghiorgis, and Aaron Aponick*

Address: Department of Chemistry, University of Florida, P.O. Box 117200, Gainesville, FL 32611, U.S.A.

Email: Aaron Aponick*- aponick@chem.ufl.edu

* Corresponding author

General procedures and characterization data for all new compounds.

General:

All reactions were carried out under an atmosphere of nitrogen unless otherwise specified. Anhydrous solvents were transferred via syringe to flame-dried glassware, which had been cooled under a stream of dry nitrogen. Anhydrous tetrahydrofuran (THF), acetonitrile, ether, dichloromethane, and pentane were dried using an MBRAUN solvent purification system. Analytical thin layer chromatography (TLC) was performed with 250 μm Silica Gel 60 F254 pre-coated plates (EMD Chemicals Inc.). Flash column chromatography was performed on 230-400 Mesh 60 Å Silica Gel (Whatman Inc.). The eluents employed are reported as volume:volume percentages. Melting points were recorded on a MEL-TEMP® capillary melting point apparatus and are uncorrected. Proton nuclear magnetic resonance (^{1}H NMR) spectra were recorded using Varian Unity Inova 500 MHz and Varian Mercury 300 MHz spectrometers. Chemical shift (δ) is reported in parts per million (ppm) downfield relative to tetramethylsilane (TMS, 0.0 ppm) or $CDCl_3$ (7.26 ppm). Coupling constants (*J*) are reported in Hz. Multiplicities are reported using the following abbreviations: s, singlet; d, doublet; t, triplet; q, quartet; m, multiplet; br, broad; Carbon-13 nuclear magnetic resonance (^{13}C NMR) spectra were recorded using a Varian Unity Mercury 300 spectrometer at 75 MHz. Chemical shifts are reported in ppm relative to the carbon resonance of $CDCl_3$ (77.00 ppm).

Infrared spectra were obtained on a Perkin Elmer Spectrum RX-1 at 0.5 cm^{-1} resolution and are reported in wave numbers. High resolution mass spectra (HRMS) were obtained by The Mass Spectrometry Core Laboratory of the University of Florida, and are reported as *m*/*e* (relative ratio). Accurate masses are reported for the molecular ion (M^+) or a suitable fragment ion. Gas Chromatography analyses were obtained using a Hewlett Packard HP 5890 Series II - FID Detector.

Compounds **7**, **8**, **10**, **11**, **12**, **13** and **19** have been described in the literature and as prepared here satisfactorily matched all previously reported data.

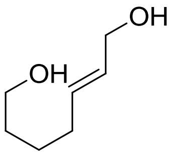

7
Ref. 1

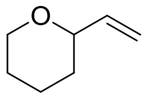

8
Ref. 4

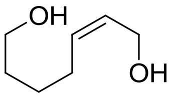

10
Ref. 2

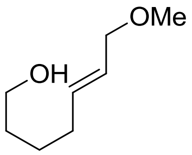

11
Ref. 2

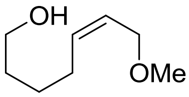

12
Ref. 2

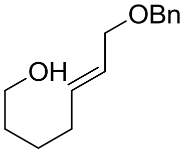

13
Ref. 3

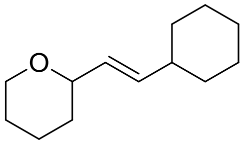

19
Ref. 5

<u>Preparation of 14 and 16:</u>

Compounds **14** and **16** were prepared in two steps from (*E*)-7-(tetrahydro-2*H*-pyran-2-yloxy)hept-2-en-1-ol; protection of the allyl alcohol with 3 equivalents of TBDPSCl or BzCl in the presence of 3 equivalents of Et_3N in CH_2Cl_2 at room temperature, followed by deprotection of the terminal non-allylic alcohol using 10 mol% of PPTS in MeOH at room temperature.

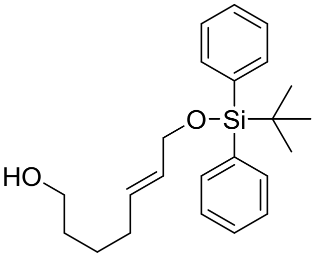

(*E*)-7-(*tert*-butyldiphenylsilyloxy)hept-5-en-1-ol (14).
Colorless oil; R_f = 0.30 (30% EtOAc/hexanes); IR (neat) 3343, 2932, 2857, 1471, 1462, 1427, 1112, 1055, 969 cm^{-1}; ^{1}H NMR (500 MHz, $CDCl_3$): δ 7.69-7.66 (m, 4H), 7.42-7.36 (m, 6H), 5.65 (dt, *J* = 15.0, 6.5 Hz, 1H), 5.55 (dt, *J* = 15.0, 5.5 Hz, 1H) 4.16 (d, *J* = 5.5 Hz, 1H), 3.64 (t, *J* = 6.5 Hz, 2H), 2.05 (q, *J* = 7.0 Hz, 2H), 1.59-1.41 (m, 4H), 1.29 (bs, 1H), 1.05 (s, 9H); ^{13}C NMR (75 MHz, $CDCl_3$): δ 135.8, 134.2, 131.1, 129.8, 129.4, 127.8, 64.9, 63.1, 32.5, 32.2, 27.1, 25.6, 19; HRMS (ESI) Calcd for $C_{23}H_{32}NaO_2Si$ $(M+Na)^+$: 391.2064; found 391.2082.

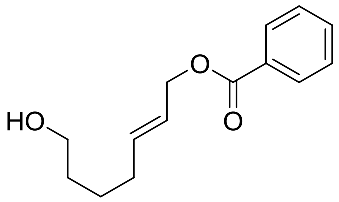

(*E*)-7-hydroxyhept-2-enyl benzoate (16).
Colorless oil; R_f = 0.18 (30% EtOAc/hexanes); IR (neat) 3390, 2936, 2862, 1418, 1452, 1273, 113, 1070, 1026, 973, 712 cm^{-1}; ^{1}H NMR (300 MHz, $CDCl_3$): δ 8.01 (d, J = 6.3 Hz, 2H), 7.53-7.36 (m, 3H), 5.82 (dt, J = 15.6, 6.3 Hz, 1H), 5.65 (dt, J = 15.3, 6.9 Hz, 1H), 4.72 (d, J = 6.0 Hz, 2H), 3.60 (t, J = 6.3 Hz, 2H), 2.08 (q, J = 6.9 Hz, 2H), 1.71 (bs, 1H), 1.60-1.41 (m, 4H); ^{13}C NMR (75 MHz, $CDCl_3$): δ 166.6, 136.2, 133.1, 130.5, 129.8, 128.5, 124.4, 65.8, 62.8, 32.3, 32.1, 25.2; HRMS (ESI) Calcd for $C_{14}H_{18}NaO_3$ $(M+Na)^+$: 257.1148; found 257.1152.

<u>Preparation of 15:</u>

Compound **15** was prepared in two steps from (*E*)-7-(*tert*-butyldimethylsilyloxy)hept-2-en-1-ol [7]; protection of the allyl alcohol using 3 equivalents of 3,4-dihydro-2*H*-pyran and 10 mol % of PPTS in CH_2Cl_2 at room temperature, followed by deprotection of the terminal non-allylic alcohol with 2 equivalents of TBAF in THF at room temperature.

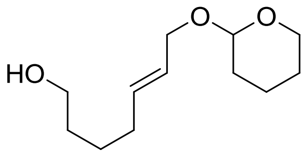

(*E*)-7-(tetrahydro-2H-pyran-2-yloxy)hept-5-en-1-ol (15).
Colorless oil; R_f = 0.35 (30% EtOAc/hexanes); IR (neat) 3410, 2938, 2864, 1117, 1075, 1023, 970 cm^{-1}; ^{1}H NMR (500 MHz, $CDCl_3$): δ 5.73 (dt, *J* = 15.5, 7.0 Hz, 1H), 5.59 (dt, *J* = 15.5, 7.0 Hz, 1H), 4.63 (dd, *J* = 4.0, 3.0 Hz, 1H), 4.19 (ddq, *J* = 12.0, 5.5, 1.0 Hz, 1H), 3.92 (dd, *J* = 12.0, 7.0 Hz, 1H), 3.87 (dd, *J* = 8.5, 5.0 Hz, 1H), 3.64 (t, *J* = 6.5 Hz, 2H), 3.52-3.48 (m, 1H), 2.09 (q, *J* = 7.0 Hz, 2H) 1.86-1.36 (m, 11H); ^{13}C NMR (75 MHz, $CDCl_3$): δ 134.3, 126.7, 98.0, 68.0, 63.0, 62.4, 32.4, 32.2, 30.9, 25.7, 25.4, 19.8; HRMS (ESI) Calcd for $C_{11}H_{22}NaO_3$ $(M+Na)^+$: 237.1467; found 237.1463.

<u>Preparation of 17 and 18:</u>

Compounds **17** and **18** were prepared in three steps from 2-(hex-5-ynyloxy)tetrahydro-2*H*-pyran (**17.1**) [6].

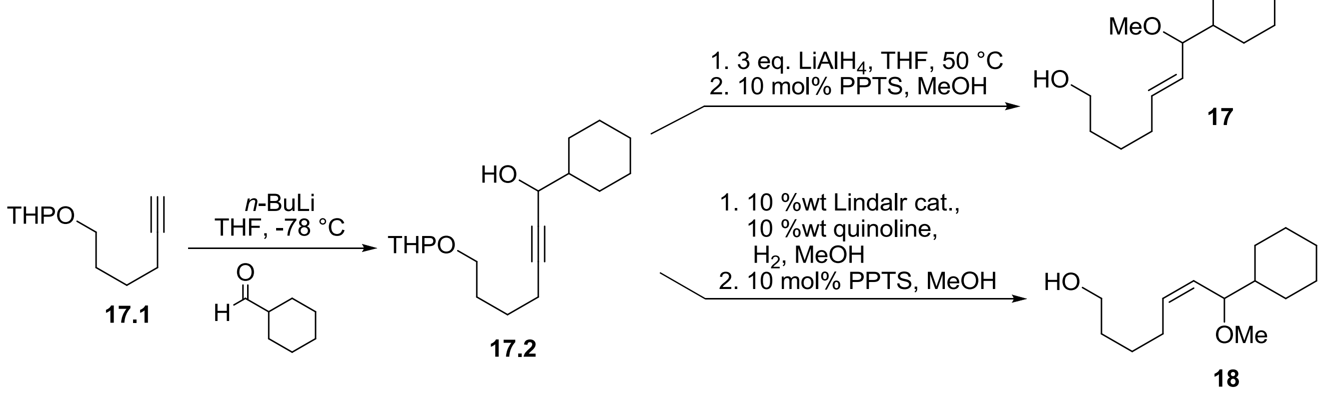

.

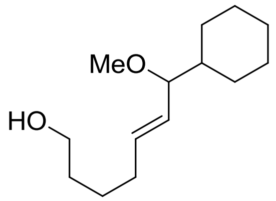

(*E*)-7-cyclohexyl-7-methoxyhept-5-en-1-ol (17).
Colorless oil; R_f = 0.28 (30% EtOAc/hexanes); IR (neat) 3402, 2928, 2853, 1450, 1095, 972 cm^{-1}; ^1H NMR (300 MHz, $CDCl_3$): δ 5.55 (dt, *J* = 15.3, 7.0 Hz, 1H), 5.25 (dd, *J* = 15.3, 8.1 Hz, 1H), 3.64 (t, *J* = 6.0 Hz, 2H), 3.22 (s, 3H), 3.17 (t, *J* = 7.7 Hz, 1H), 2.10 (q, *J* = 7.0 Hz, 2H), 1.94-1.83 (m, 2H), 1.73-0.89 (m, 14H); ^{13}C NMR (75 MHz, $CDCl_3$): δ 134.7, 129.4, 87.6, 62.8, 56.2, 42.6, 32.4, 32.2, 29.5, 29.0, 26.8, 26.3, 26.3, 25.7; HRMS (ESI) Calcd for $C_{14}H_{26}NaO_2$ $(M+Na)^+$: 249.1825; found 249.1832.

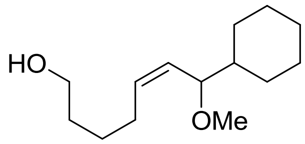

(*Z*)-7-cyclohexyl-7-methoxyhept-5-en-1-ol (18).
Colorless oil; R_f = 0.30 (30% EtOAc/hexanes); IR (neat) 3375, 2924, 2852, 1450, 1085, 970 cm^{-1}; ^1H NMR (300 MHz, $CDCl_3$): δ 5.64 (dt, *J* = 11.1, 7.5 Hz, 1H), 5.20 (dd, *J* = 11.1, 9.6 Hz, 1H), 3.68-3.59 (m, 3H), 3.21 (s, 3H), 2.17-2.02 (m, 2H), 1.91-0.88 (m, 16H); ^{13}C NMR (75 MHz, $CDCl_3$): δ 134.0, 129.7, 81.1, 63.0, 56.2, 43.0, 32.6, 29.5, 28.8, 27.8, 26.9, 26.4, 26.1; HRMS (ESI) Calcd for $C_{14}H_{26}NaO_2$ $(M+Na)^+$: 249.1825; found 249.1835.

<u>General procedure for the Au-catalyzed cyclization:</u>

A solution of *n*-decane (0.15 mmol) and the substrate (0.3 mmol) in dry CH_2Cl_2 (1 mL) was added in one portion at room temperature to an aluminum foil covered 5 mL vial containing a solution of (acetonitrile)[(*o*-biphenyl)di-*tert*-butylphosphine]gold(I) hexafluoroantimonate (11.6 mg, 0.015 mmol, 5 mol%) in dry CH_2Cl_2 (0.5 mL) and activated MS-4Å (70 mg) under a N_2 atmosphere. The reaction was monitored by taking 25 μL aliquots which were immediately diluted in 400 μL of dry CH_2Cl_2 containing 15-20 mg of beads QuadraPure™ MPA. A 1 μL aliquot of this solution was analyzed by gas chromatography.

<u>Determination of conversion of 8:</u>

The conversion was determined by gas chromatography analysis of 2-vinyltetrahydro-2*H*-pyran **8** and *n*-decane. A calibration plot had been made using known quantities of **8** and *n*-decane (Figure S-1).

Column: RESTEK Rtx®-5 (Crossbond 5% diphenyl – 95% dimethyl polysiloxane), 30 meters, 0.25 mm ID, 0.5 μm df.

Temperature: 60 °C for 3 min, 10 °C increase → 110 °C, 40 °C increase → 275 °C, 275 °C for 2 min.

Time: t_R (**8**): 5.1 min; t_R (*n*-decane): 7.4 min.

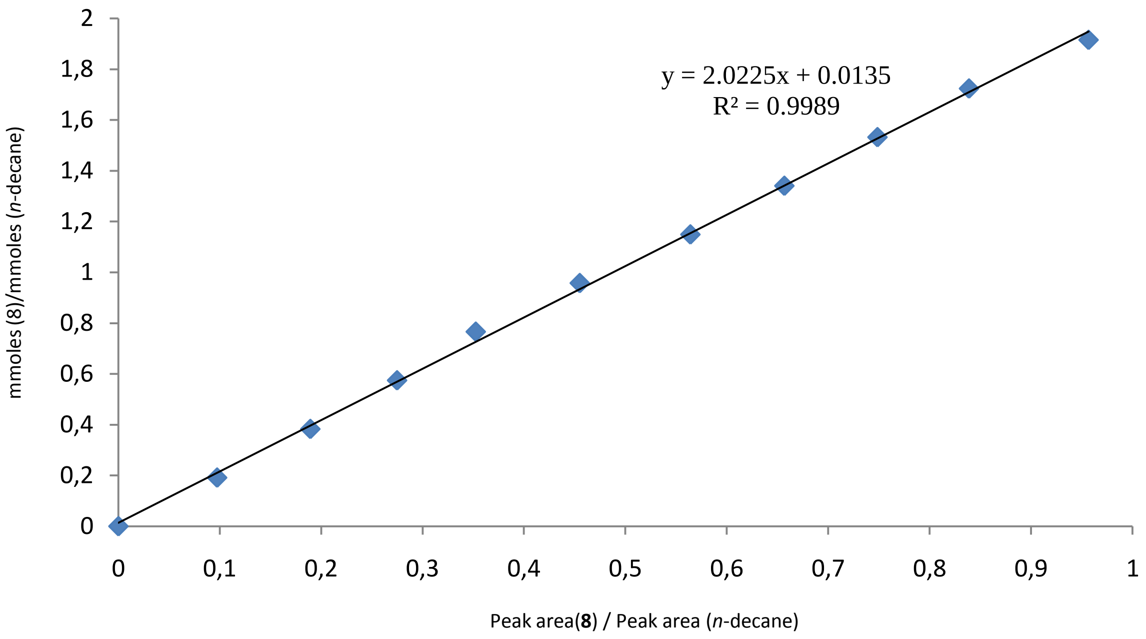

Figure S-1. Calibration plot of **8** *vs n*-decane.

Determination of conversion of 19:

The conversion was determined by gas chromatography analysis of (*E*)-2-(2-cyclohexylvinyl)tetrahydro-2*H*-pyran **19** and *n*-decane. A calibration plot had been made using known quantities of **19** and *n*-decane (Figure S-2).

Column: RESTEK Rtx®-5 (Crossbond 5% diphenyl – 95% dimethyl polysiloxane), 30 meters, 0.25 mm ID, 0.5 µm df.

Temperature : 60 °C for 3 min, 10 °C increase → 110 °C, 20 °C increase → 275 °C, 275 °C for 8 min.

Time: t_R (*n*-decane): 7.4 min; t_R (**19**): 13.4 min.

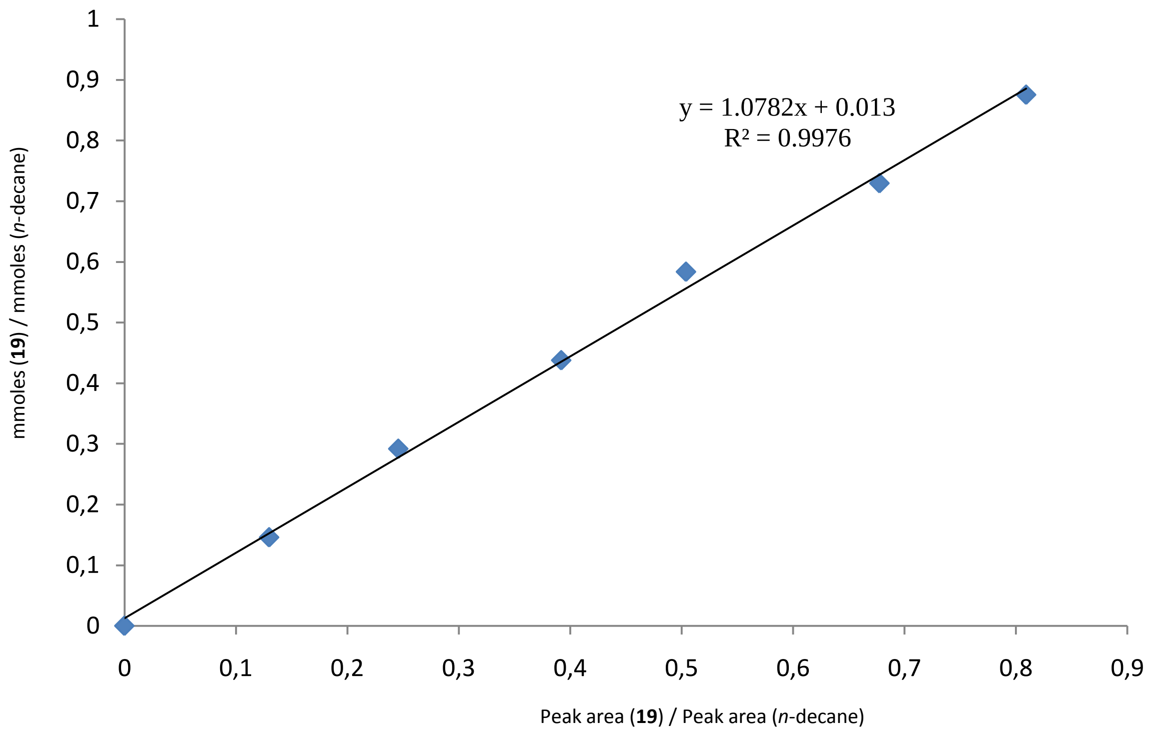

Figure S-2. Calibration plot of **19** *vs* *n*-decane.

References:

1. Tanaka, S.; Seki, T.; Kitamura, M. *Angew. Chem. Int. Ed*. **2009**, *48*, 8948-8951.
2. Harms, A. E.; Stille, J. R. *Organometallics* **1994**, *13*, 1456-1464.
3. Takacs, J. M.; Myoung, Y.-C.; Anderson, L. G. *J. Org. Chem.* **1994**, *59*, 6928-6942.
4. Froestl, W.; Mickel, S. T.; von Sprecher, G.; Diel, P. J.; Hall, R. G.; Maier, L.; Strub, D.; Melillo, V.; Baumann, P. A. *J. Med. Chem.* **1995**, *38*, 3313-3331.
5. Aponick, A.; Li, C.-Y.; Biannic, B. *Org. Lett.* **2008**, *10*, 669-671.
6. Marino, J. P.; Nguyen, H. N. *J. Org. Chem.* **2002**, *67*, 6291-6296.
7. Frankowski, K. J.; Golden, J. E.; Zeng, Y.; Lei, Y.; Aube, J. *J. Am. Chem. Soc.* **2008**, *130*, 6018-6024.

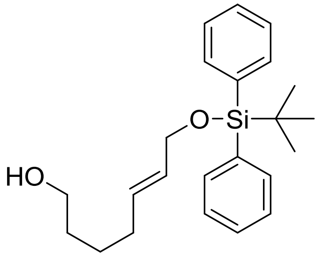
HO
O
Si

14

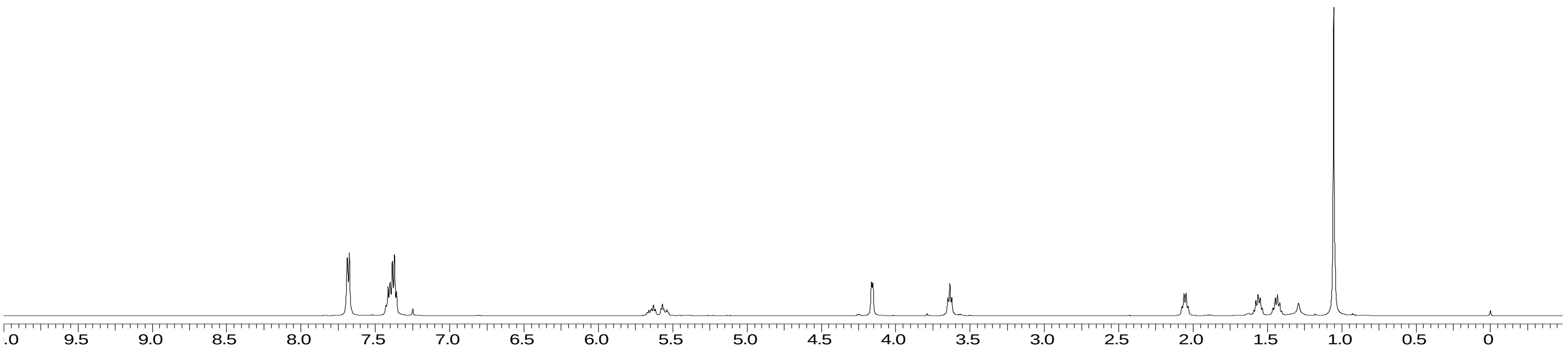
.0
9.5
9.0
8.5
8.0
7.5
7.0
6.5
6.0
5.5
5.0
4.5
4.0
3.5
3.0
2.5
2.0
1.5
1.0
0.5
0

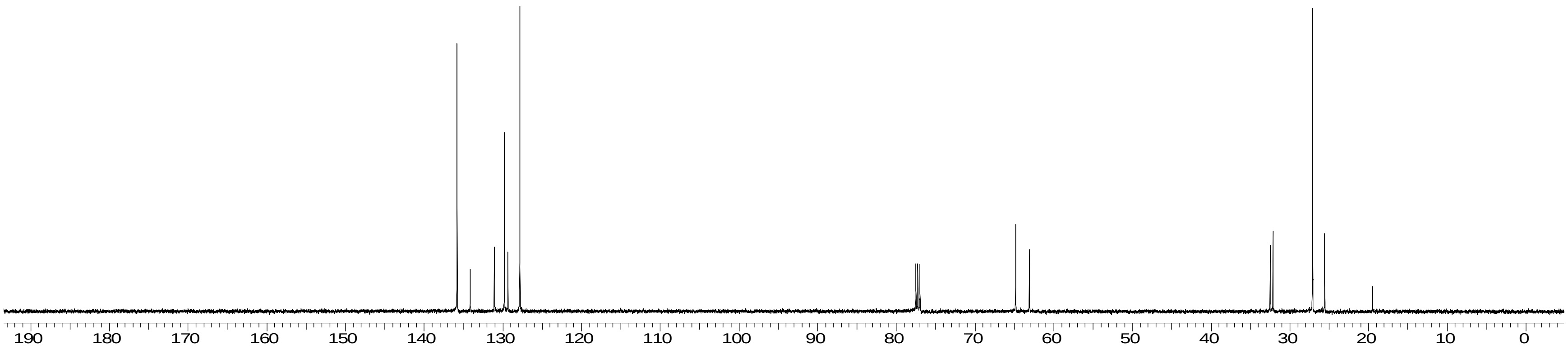
190
180
170
160
150
140
130
120
110
100
90
80
70
60
50
40
30
20
10
0

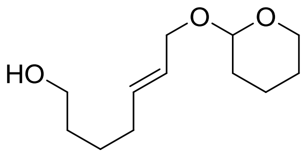

15

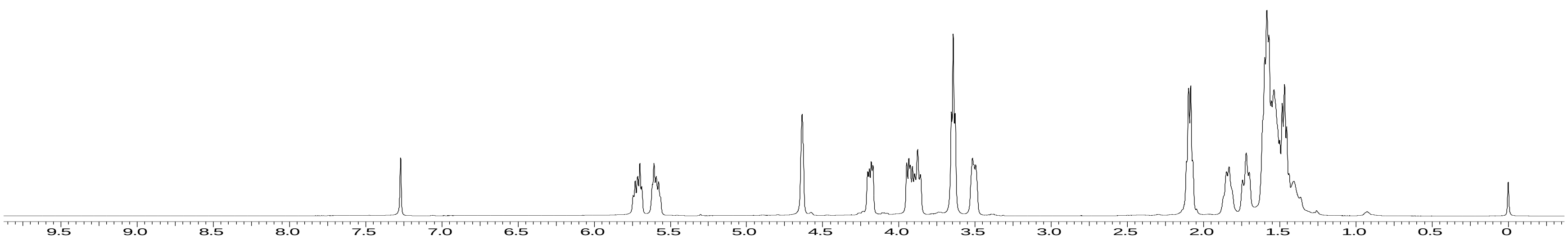

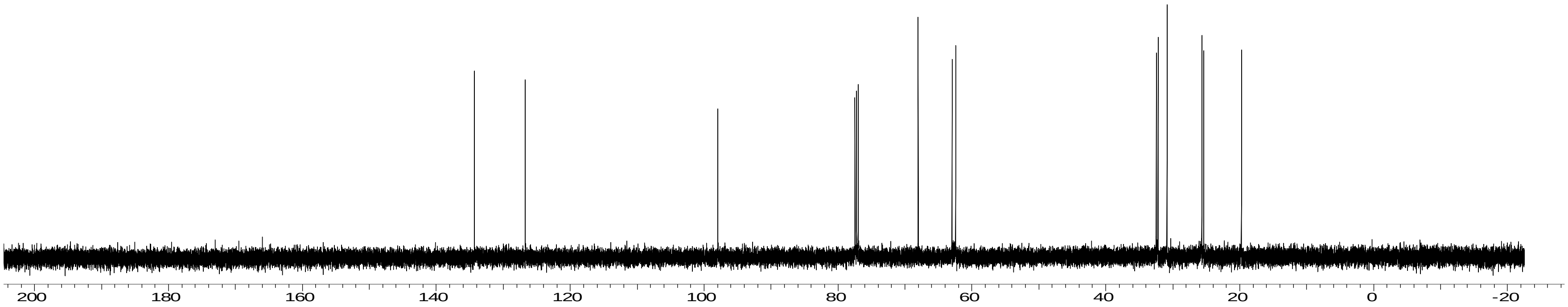

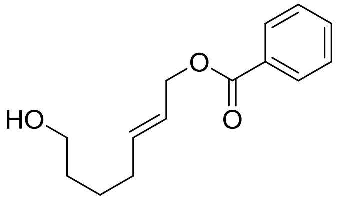
HO
O
O

16

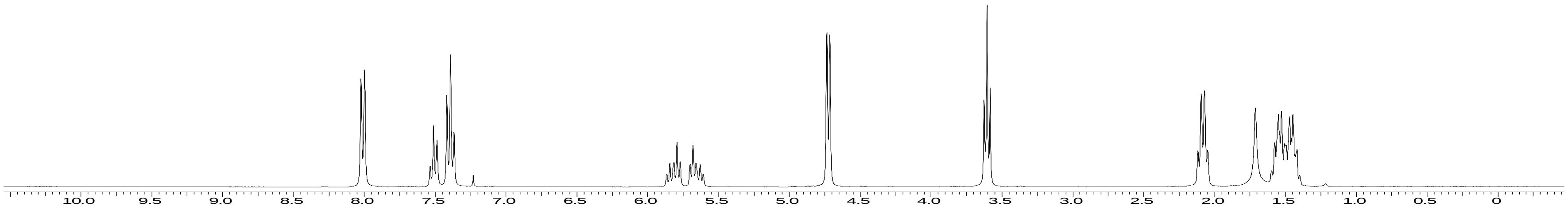
10.0 9.5 9.0 8.5 8.0 7.5 7.0 6.5 6.0 5.5 5.0 4.5 4.0 3.5 3.0 2.5 2.0 1.5 1.0 0.5 0

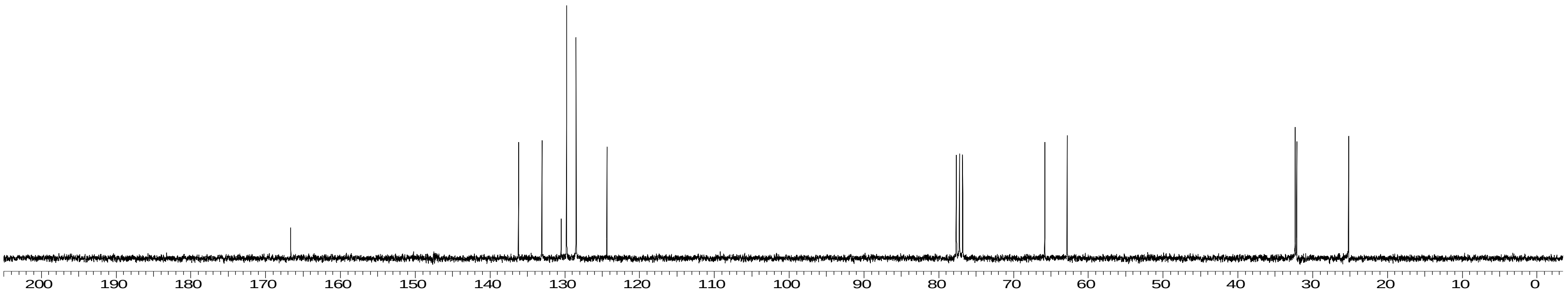
200 190 180 170 160 150 140 130 120 110 100 90 80 70 60 50 40 30 20 10 0

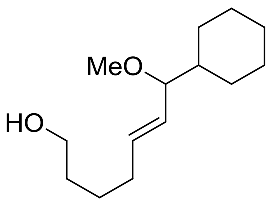
MeO
HO

17

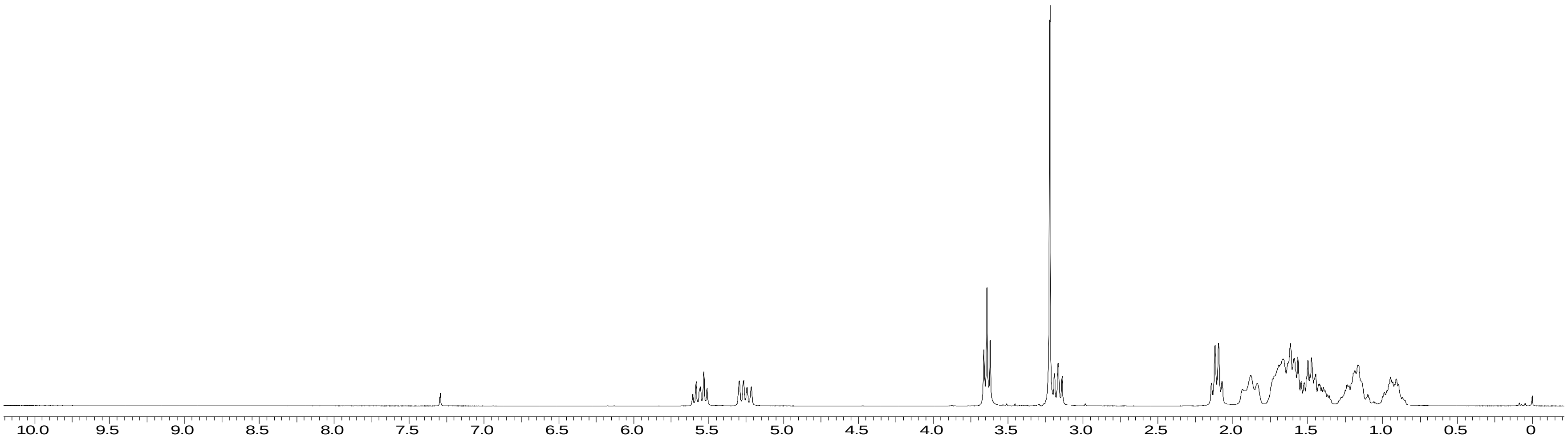
10.0 9.5 9.0 8.5 8.0 7.5 7.0 6.5 6.0 5.5 5.0 4.5 4.0 3.5 3.0 2.5 2.0 1.5 1.0 0.5 0

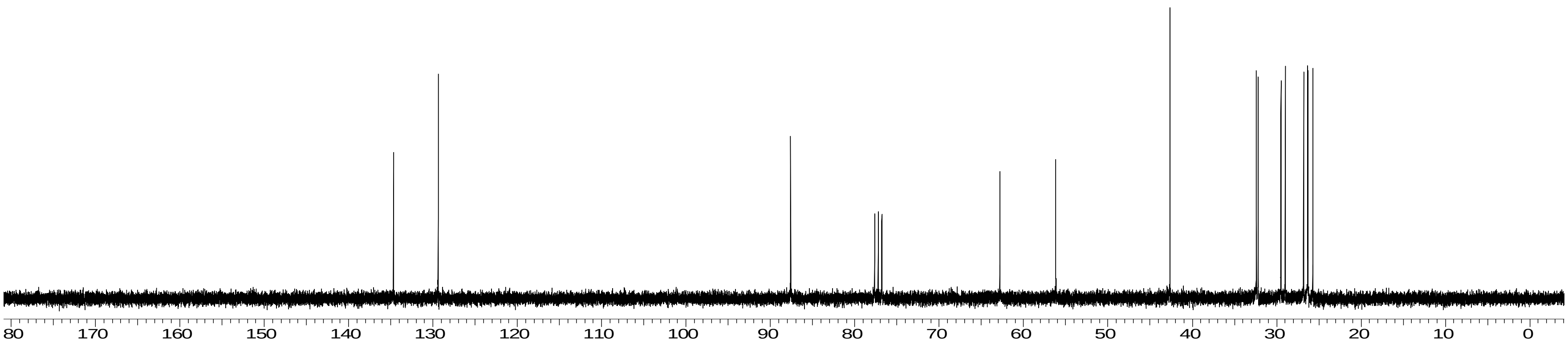
80 170 160 150 140 130 120 110 100 90 80 70 60 50 40 30 20 10 0

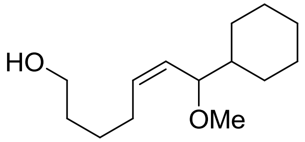
HO
OMe

18

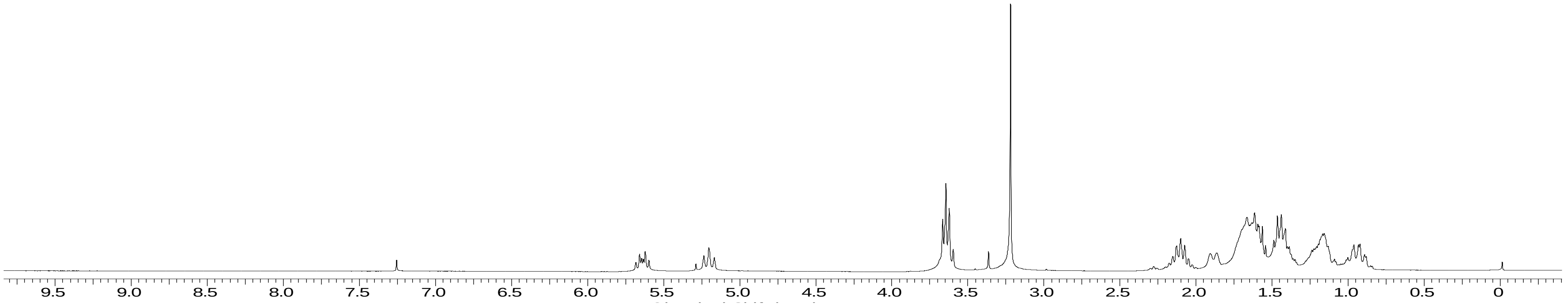
9.5
9.0
8.5
8.0
7.5
7.0
6.5
6.0
5.5
5.0
4.5
4.0
3.5
3.0
2.5
2.0
1.5
1.0
0.5
0

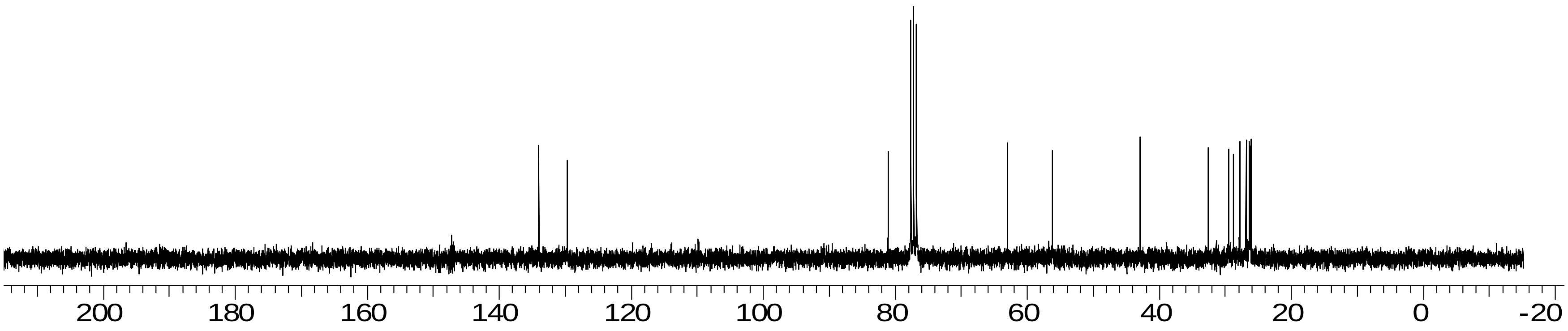
200
180
160
140
120
100
80
60
40
20
0
-20
